# Unveiling Integrated Functional Pathways Leading to Enhanced Respiratory Disease Associated With Inactivated Respiratory Syncytial Viral Vaccine

**DOI:** 10.3389/fimmu.2019.00597

**Published:** 2019-03-29

**Authors:** Marsha S. Russell, Marybeth Creskey, Abenaya Muralidharan, Changgui Li, Jun Gao, Wangxue Chen, Louise Larocque, Jessie R. Lavoie, Aaron Farnsworth, Michael Rosu-Myles, Anwar M. Hashem, Carole L. Yauk, Jingxin Cao, Gary Van Domselaar, Terry Cyr, Xuguang Li

**Affiliations:** ^1^Centre for Biologics Evaluation, Biologics and Genetic Therapies Directorate, Health Products and Food Branch (HPFB), Health Canada and WHO Collaborating Center for Standardization and Evaluation of Biologicals, Ottawa, ON, Canada; ^2^Department of Biochemistry, Microbiology and Immunology, Faculty of Medicine, University of Ottawa, Ottawa, ON, Canada; ^3^National Institutes for Food and Drug Control, WHO Collaborating Center for Standardization and Evaluation of Biologicals, Beijing, China; ^4^Human Health Therapeutics Research Centre, National Research Council Canada, Ottawa, ON, Canada; ^5^Immunotherapy Unit, Department of Medical Microbiology and Parasitology, Faculty of Medicine and Vaccines, King Fahd Medical Research Center, King Abdulaziz University, Jeddah, Saudi Arabia; ^6^Mechanistic Studies Division, Environmental and Radiation Health Sciences Directorate, Healthy Environments and Consumer Safety Branch (HECSB), Health Canada, Ottawa, ON, Canada; ^7^National Microbiology Laboratory, Public Health Agency of Canada, Winnipeg, MB, Canada

**Keywords:** RSV vaccines, RSV vaccine-enhanced disease, proteomics, systems biology, hypercoagulation, cytokine, mechanistic studies

## Abstract

Respiratory syncytial virus (RSV) infection is a severe threat to young children and the elderly. Despite decades of research, no vaccine has been approved. Notably, instead of affording protection, a formalin-inactivated RSV vaccine induced severe respiratory disease including deaths in vaccinated children in a 1960s clinical trial; however, recent studies indicate that other forms of experimental vaccines can also induce pulmonary pathology in pre-clinical studies. These findings suggest that multiple factors/pathways could be involved in the development of enhanced respiratory diseases. Clearly, a better understanding of the mechanisms underlying such adverse reactions is critically important for the development of safe and efficacious vaccines against RSV infection, given the exponential growth of RSV vaccine clinical trials in recent years. By employing an integrated systems biology approach in a pre-clinical cotton rat model, we unraveled a complex network of pulmonary canonical pathways leading to disease development in vaccinated animals upon subsequent RSV infections. Cytokines including IL-1, IL-6 GRO/IL-8, and IL-17 in conjunction with mobilized pulmonary inflammatory cells could play important roles in disease development, which involved a wide range of host responses including exacerbated pulmonary inflammation, oxidative stress, hyperreactivity, and homeostatic imbalance between coagulation and fibrinolysis. Moreover, the observed elevated levels of MyD88 implicate the involvement of this critical signal transduction module as the central node of the inflammatory pathways leading to exacerbated pulmonary pathology. Finally, the immunopathological consequences of inactivated vaccine immunization and subsequent RSV exposure were further substantiated by histological analyses of these key proteins along with inflammatory cytokines, while hypercoagulation was supported by increased pulmonary fibrinogen/fibrin accompanied by reduced levels of plasma D-dimers. Enhanced respiratory disease associated with inactivated RSV vaccine involves a complex network of host responses, resulting in significant pulmonary lesions and clinical manifestations such as tachypnea and airway obstruction. The mechanistic insight into the convergence of different signal pathways and identification of biomarkers could help facilitate the development of safe and effective RSV vaccine and formulation of new targeted interventions.

## Introduction

Respiratory syncytial virus (RSV) is the most frequent cause of serious respiratory illness in infants, the elderly and immunocompromised adults (1). Despite decades of research, no vaccine has been licensed. The slow progress in RSV vaccine development is partially due to the unusual disease associated with a formalin-inactivated RSV vaccine (FI-RSV) which caused vaccine-associated enhanced respiratory disease (VERD), with two fatalities and hospitalization of 80% of vaccine recipients in a clinical trial in the 1960s (2, 3). While formalin, the inactivation agent for the RSV vaccine preparation, might play a role in VERD development, recent studies suggest that other forms of RSV vaccines also induce VERD, suggesting multiple pathways could be involved in this phenomenon. Clearly, along with the uncovering of additional immunological correlates (4–7), increasing research activities toward better understanding of VERD is of critical importance to develop safe and effective RSV vaccine (8, 9).

As of today, there are at least 25 ongoing RSV vaccine trials in the Unites States and 15 registered trials at the WHO, with provision of data on VERD investigation of candidate vaccines being one of the regulatory requirements prior to progression into clinical trials (10). Moreover, it is recommended that vaccine candidates eliciting high levels of IL-4 and/or IL-13 and/or inducing non-neutralizing antibodies in pre-clinical studies be excluded for progression into clinical trials for seronegative individuals (11). As mentioned earlier, it is important to note that VERD is not exclusive to formalin inactivated vaccines. Indeed, chimeric RSV FG glycoprotein (12), recombinant vaccinia virus RSV-G (9), low RSV F doses of post-fusion F (13), ultra violet inactivated RSV, and purified fusion protein (14), along with ring-nanostructures formed by the recombinant nucleoprotein *N* of hRSV (15), have all resulted in VERD following RSV infection. The exponential rise in vaccine candidates in a variety of platforms for RSV targeted to seronegative infants presents new challenges within the field. Moreover, a surge of novel platforms for immunizations stresses the need for authenticated biomarkers and animal models to minimize the risk for VERD before novel RSV vaccines reach seronegative infants (16).

Much research has been done to understand this VERD phenomenon. It is known that following FI-RSV immunization and subsequent infection with RSV, there is a skewed TH2 dominant response (17), accompanied by increased serum antibodies (Ab) against RSV with low neutralizing ability (18–20), and poor RSV specific IgG avidity (14) as reviewed in (21). Although antibody avidity has been shown to be a hallmark of FI-RSV induced VERD in the mouse model, in cotton rats and human infants it has been shown that avidity is not a contributing factor (22). In animal models, deficiencies in TLR stimulation (17) and the involvement of CD4 and CD8 T cells in mediating VERD have also been observed (21, 23, 24). It is of note that one group has taken a holistic approach to identify biomarkers associated with ERD, with the study conducted in mice using a vaccinia virus vectored vaccine (9). These investigators found that some elevated proteins related to the influx or homing of eosinophils and neutrophils into the lungs of the VERD animals. It would be of interest to use a systems biology approach to investigate VERD in cotton rat (*Sigmodon hispidus*), the arguably more ideal animal model since it appears to be the only animal model that best mirrors the human autopsy findings (21, 25).

Cotton rats are 50-to-100-fold more permissive than mice to infection by RSV (26) and unlike mice, exhibit VERD pathology similar to that in human autopsies (19, 25, 27). Moreover, this model was valuable in pre-clinical evaluation of the prophylactic antibodies (28, 29) currently being used in human infants. Initial autopsy results conducted on the lungs of the infants showed peribronchiolar monocytic infiltration dominated with eosinophils (2, 3). However, a more in-depth evaluation of the autopsy tissue by Prince et al. uncovered the majority of the peribronchial infiltrates were comprised of lymphocytes along with some neutrophils and macrophages. As well, the majority of the bronchial exudate was comprised of neutrophils with some macrophages and lymphocytes. Only 1–2% of the cellular infiltrate was eosinophils in both infants (25). Cotton rats vaccinated twice with FI-RSV and subsequently infected with RSV display VERD with marked bronchiolitis and alveolitis with predominant neutrophil infiltration into alveolar space, with alveolitis peaking 5 days post-RSV challenge (27). Moreover, similar to humans, immunity induced by natural infection in cotton rats is short-lived (30). While others have measured some of the cytokine profile in FI-RSV VERD in cotton rats, the breadth of the immune response has not been evaluated (17). Increasingly, vaccine candidates progressing to clinical trials are based on data conducted in the cotton rat animal model (31, 32). However, little is known about the molecular mechanisms underlining VERD pathogenesis in this model. Clearly, it would be of value to investigate the mechanisms of VERD using the cotton rat (33). Herein, we used a systems biology approach to investigate functional pathways involved in VERD in cotton rats. We conducted paired analysis of pulmonary function and histopathological measures with global proteomic profiling to study the mechanisms underlying RSV-induced VERD. Our findings provide mechanistic insight into the vaccine-induced disease enhancement.

## Methods

### Animals and Ethics Statement

Six to seven week old cotton rats were obtained from Envigo, Somerset, N.J., USA. All animal procedures were approved by the Health Canada Ottawa Animal Care Committee and performed in accordance with institutional guidelines.

### Virus, Immunization, and Infection

RSV A2 strain (ATCC VR-1540, Manassas, Va) was propagated in Hep-2 cells (ATCC CCL-23, Manassas, Va) cultivated in growth media (Dulbecco's Modified Eagle Medium supplemented with 1.5 g/sodium bicarbonate, 2 mM Glutamax, 1 mM HEPES, 20 U/ml Penicillin, 0.02 mg/ml Streptomycin, and 10% FBS). Viral preparations were made by infecting Hep-2 cells at a multiplicity of 0.02 followed by 2 h absorption at room temperature. Monolayers were then washed once and cultured in serum-free medium until 80% cytopathic effect was reached. Culture media was harvested, centrifuged at 1,000 g for 10 min at 4°C, filtered (0.45 μm), and then centrifuged at 62,000 g for 30 min at 4°C. The resulting pellet was resuspended in HBSS with 25 mM HEPES buffer.

FI-RSV was prepared as previously described (25). Briefly, Hep-2 cells were infected as with RSV viral preparations and an aliquot taken for viral titer determination. Formalin was added 1:4,000 and incubated at 37°C for 3 days and then ultracentrifuged 62,000 g for 30 min. Pellets were resuspended to 1/25th of the original volume in DMEM medium without supplements and adsorbed to aluminum hydroxide (4 mg/ml). The resulting material was centrifuged at 1,000 rpm for 10 min and resuspended to 1/4th volume in DMEM resulting in a final vaccine that was concentrated 100-fold and contained 16 mg/ml alum. FI-Mock vaccine was prepared similarly alongside without virus.

On day 0 and 21, FI-RSV and FI-Mock groups were intramuscularly vaccinated at 1 × 10^6^ PFU while the PBS group was given PBS buffer. The RSV group infection was intranasally vaccinated with live RSV virus at 1 × 10^6^ PFU. On day 49, all animals were challenged intranasally with RSV at 1 × 10^6^PFU. Five days post-challenge, the animals were sacrificed. Lungs from the same animal were used for viral titration as well as histology. In order to do this, one lobe used of the lung was used for virus titration and the other lobe was fixed in 10% neutral buffered formalin (Sigma) under 25 cm of water pressure. For the lungs used for proteomics, the aorta and the caudal vena cava were clamped. A 25G needle was placed in the right ventricle and a puncture was made in the left ventricle to allow for drainage. The PBS-Heparin mixture was pumped through at 10 mL/min using a Masterflex L/S digital pump system (Cole-Parmer). The lobes of the lungs were removed and snap frozen in liquid nitrogen.

### Pulmonary Functional Assessment

Lung function was evaluated utilizing unrestrained whole-body plethysmography from day 0 to 5 post-challenge. Enhanced pause (Penh) was measured using a whole-body plethysmograph (Buxco Electronics, Wilmington, NC) as previously described (34). Briefly, one cotton rat was placed within each plethysmograph chamber and following a 15 min acclimation, breaths per minute (bpm) and Penh were calculated based on pressure and volume changes over 5 min. Box humidity and temperature was set at that recorded within the room; and body temperature was set at 39.5°C. Bias flow was set at 2 l/min, resulting in a flow past the animals of 1.2 ml/s. The instrument software calculated Pause, and Penh, a dimensionless measure that combines both time and flow rates to describe ventilation. Penh has been connected to alterations in baseline respiratory patterns (35) and validated as an indication of airway obstruction (36). Penh has been used as an indicator of airway obstruction in RSV infections in mice (23) and both mouse and cotton rat influenza infections (34, 37).

### Histology

Half of the lung was harvested 5 days following RSV challenge as described above and stained with H&E. A certified veterinary pathologist performed the scoring in a manner masked to experiment groups. Each sample was assessed for peribronchiolitis and alveolitis and given a pathology score as follows: 0—normal, within naïve parameters; 1—mild peribronchiolitis/alveolitis with peribronchiolitis detected in 1 event/section (event = bronchiole showing lymphocytic infiltration) and alveolitis in < 10% area of section; 2-moderate peribronchiolitis/alveolitis with peribronchiolitis detected in 2–3 events/section and alveolitis in 20–50% area of section; 3-severe peribronchiolitis/alveolitis with peribronchiolitis detected in 4 or more events/section and alveolitis in >50% area of section.

### Lung Viral Titration

Tissue for lung viral titration was obtained as described above. Half the lung was weighed and homogenized. 10-fold serial dilutions were performed and incubated on Hep-2 cells in 6-well plates for 2 h at 37° C. Plates were rocked every 15 min and overlaid with a 1:1 mixture of Hep-2 growth medium and 0.8% SeaKem ME agarose (Lonza Rockland ME). Following 7 days of incubation at 37° C, 5% CO_2_, plates were stained with a 1:1 mixture of DMEM and 1% agarose containing 0.2 mg/ml neutral red (Sigma-Aldrich). Plaques were counted after 24 h.

### Microneutralization Assay

Serum samples were heat-inactivated at 56°C for 30 min. Ten-fold serial dilutions from 1:10 were prepared in Hep-2 growth media and mixed with an equal volume of RSV at 16,000 PFU/ml (800 PFU/well). Virus only, serum only, or no virus (1:10 dilution serum), and cells only controls were included. Serum/virus mixtures were incubated for 1 h at 37°C, 5% CO_2_. Overnight Hep2 monolayers were infected with 100 μl/well of the serum/virus mixture in duplicate and incubated for 72 h at 37°C, 5% CO_2_.

Hundred percent ice cold methanol fixed cells were blocked with 200 μl/well blocking buffer (5% milk in PBS-0.1% tween-20) for 2 h at 37°C. Plates were washed with wash buffer (PBS-0.1% tween-20) and incubated at 37°C for 1 h with 100 μl/well anti-RSV horseradish peroxidase-labeled antibody (Meridian Life Science Inc., B65840G) 0.5 μg/ml in blocking buffer. Plates were washed and incubated for 20 min in the dark with 100 μl/well 3,3′5,5′-tetramethylbenzidine substrate (New England Bio Labs). The reaction was stopped with Stop solution (New England Bio Labs) and absorbance was read at 450 nm on a BioTek Synergy 2 plate reader. The cell control absorbance was subtracted from the serum and virus control absorbance. The serum controls were then subtracted from the diluted serum. The diluted test serum duplicates were averaged and the percentage inhibition of infection calculated as follows:

$$\begin{array}{r} \%\text{inhibition of infection}=100-\left[{\left(\text{a}-\;\text{b}\right)}^{{\;}^{*}}100\right] \end{array}$$

Where a = average test serum dilution absorbance, and b = average virus only absorbance.

### Enzyme Linked Immunosorbant Assay (ELISA)

Serum from immunized and challenged cotton rats were collected for determination of IgG levels. Ninety six-well plates were coated with sucrose purified whole virus at 40,000 PFU/ml overnight at 4°C in PBS. Next day, the plates were washed and blocked with 3% BSA in PBS containing 0.05% Tween 20 for 1 h at 37°C. Serial dilutions of the cotton rat serum in blocking buffer were then added for 1 h at 37°C. After washing, HRP-conjugated goat anti-mouse IgG HRP (Southern Biotech Cat#1030-05) (which cross reacts with cotton rat) at 1:5,000 dilution in blocking buffer was added for 1 h at 37°C. The plates were again washed and Tetramethylbenzidine substrate (Cell Signaling Technology) was added for 20 min at room temperature. The reaction was then stopped with 0.16 M sulfuric acid. The plates were read spectrophotometrically at 450 nm.

### Mass Spectrometric Analysis and Data Processing

Lung tissue was homogenized (TissueLyser II, Qiagen), followed by extracted protein being reduced, alkylated, digested with trypsin, labeled with isobaric tags (TMT 10plex, Life Technologies), fractionated (OFFgel, Agilent) and peptides analyzed with Orbitrap Fusion (Thermo) by LC-SPS-MS3. Data was processed with ProteomeDiscoverer 2.1 for protein identification and quantification. SEQUEST HT algorithm searched protein databases for cotton rat (Genbank, downloaded 20150408, 601 entries), rat (Uniprot-Trembl, downloaded 20150730, 91053 entries), and common contaminants (cRAP) with ion tolerances 5 ppm (precursor), 0.6 Da (fragment), two missed cleavages, fixed modifications TMT tags on peptide *N* termini/lysine residues, carbamidomethylation of cysteine residues, variable modifications *N*-terminal acetylation, methionine oxidation, and serine/threonine/tyrosine phosphorylation. Samples were normalized on total peptide, and 10 S/N used for quantification resulting in 5,997 identified proteins and of those, 5,069 were quantified. Fold change and statistical significance between each of the FI-RSV, RSV, and FI-Mock vs. the PBS group were assessed for the 5,069 proteins. Protein abundance levels were considered significantly different if (a) the difference is statistically significant (α = 0.05, *t*-test, Welch correction) and (b) the fold change (ratio of the mean) of a protein is greater than |±1.2|. This resulted in our datasets which contained 269 differentially expressed proteins in the FI-RSV group, 536 proteins in the RSV group, and 309 proteins in the FI-Mock group, over PBS.

### Bioinformatics Pathway Analysis

Data were imported into Ingenuity Pathway Analysis (IPA) (QIAGEN Inc., https://www.qiagenbioinformatics.com/products/ingenuity-pathway-analysis/) [Build Version 470319M, Content Version 27821452 (2016-06-14)] for pathway analysis. IPA up stream regulators and downstream effects analysis are predictive based on the canonical pathways identified by the proteomics analysis of the pulmonary tissue combined with the robust and integrated databases. The IPA analysis identifies the most relevant canonical pathways such as signaling and metabolic pathways, molecular networks, and biological functions for proteins identified in our data set. Based on these, the IPA algorithm predicts the direction of downstream effects on biological and disease processes. Briefly, the proteomics final datasets were uploaded into IPA for core analysis. The significance of the association between the data set and the canonical pathway is determined by two parameters: (1) A ratio of the number of proteins from the data set that map to the pathway divided by the total number of proteins that map to the canonical pathway and (2) a *p*-value calculated using Fischer's exact test determining the probability that the association between the proteins in the data set and the canonical pathway is due to chance alone. The *p*-value (“enrichment” score) assesses which biological attributes are significantly associated with the dataset. An enrichment score of 1.3 was considered significant. The overall predicted activation state of the biological attributes is assigned a z-score (<0: inhibition, >0: activation) and those that gave a z-score >2 or <-2 were considered significant. A comparison analysis was then performed and the results are graphed by *p*-value of overlap or *z*-score.

### Immunohistochemistry

Immunostaining was carried out as previously described (38) with modifications. Following 10 min antigen retrieval by boiling in 0.01 M citrate buffer (pH = 6.0) in a micro oven, sections were blocked with Protein Block (X0909, DAKO) for 2 h. Antibodies goat anti-crIL-1β, or goat anti-crIL6 (AF1009 and AF AF561, R&D Systems), or rabbit anti-MYD88 (Novus Biologicals), or rabbit anti-ITGAM (BIOSS) or rabbit anti-fibrinogen (BIOSS) were then incubated overnight at 4°C. Subsequently, the samples were incubated with secondary antibodies followed with addition of diaminobenzidine (DAB) substrate.

For each section, 15 fields of view were counted of cells in the alveoli (×40 magnification). Counting was performed using Northern Eclipse software and thresholds set using negative controls. Results were presented as the percentage of stained cells and/or the percentage of the total area per section.

### RNA Extraction and Quantitative Real-Time PCR

Quantitative real-time PCR was conducted on an ABI Prism 7500 Fast Sequence detection system (Applied Biosystems). Primer Express (Applied Biosystems) was used to design the primers and MGB probes sequences for cotton rat GRO and β-actin which was used to normalize the data. TaqMan assay reagent kits (Applied Biosystems) were used that contain pre-standardized primers and TaqMan MGB probes for NFκB2. Total RNA was isolated from the PBS/heparin perfused lungs using the RNeasy Mini Kit (Qiagen) according to manufactures instructions. The isolated RNA was used to make cDNA using the Superscript III First-Strand Synthesis System for RT-PCR (Invitrogen) according to manufacturer's instructions. The cDNA was then subjected to quantitative PCR using the TaqMan Fast Advanced Master Mix (Applied Biosystems) according to manufactures instructions. Samples were run in duplicate and C_t_ values were obtained. Fold change over PBS was calculated using the 2^−ΔΔ*CT*^ method of relative quantification (39) using β-actin as the housekeeping reference gene.

### Martius Scarlet Blue Stain for Fibrin

Martius Scarlet Blue (MSB) Stain is a trichrome stain used to visualize fibrin paraffin embedded lung tissue samples for other respiratory diseases (40). MSB was carried out as described (41), with the orcein step removed and Weiget's iron hematoxylin used for nuclear staining. Briefly, sections were prepared from the formalin-fixed and paraffin-embedded specimen. Following deparaffinization and rehydration, samples were treated with Bouin's fluid at 56°C for 1 h. Specimens were rinsed with distilled water then stained with Weigert's iron hematoxylin followed by rinsing in 95% ethanol. Specimens were then stained with Martius yellow solution containing phosphotungstic acid in 95% ethanol for 3 min, rinsed with distilled water and stained with Crystal Scarlet in 2.5% glacial acetic acid for 10 min. Specimens were then differentiated with phosphotungstic acid by 4 × 5 min washes and placed in methyl blue for 5 min. Samples were then washed with distilled water and rehydrated though ethanol and xylene and mounted. The results will stain nuclei blue, erythrocytes yellow, muscle red, collagen blue, Fibrin (early deposit) yellow (mature deposit) red and (very old deposit) blue.

### Coagulation Assay

Plasma D-dimer levels were measured at Cornell Universities Animal Health Diagnostic Centers Comparative Coagulation Section using enzyme-linked immunosorbent assay (Diagnostica Stago) using a human standard. Whole blood was collected via the abdominal aorta during necropsy and immediate mixed in a 9 parts blood to 1 part citrate 4% w/v (Sigma). The blood/citrate mix was then centrifuged at room temperature, 14,000 g for 1 min and the platelet free citrated plasma was then immediately stored at −80°C.

### Statistical Analysis

Statistical analyses were performed using GraphPad Prism software (GraphPad Software, San Diego, CA). Data was compared using a one-way ANOVA between FI-RSV and the respective control group to determine if there was a statistical significance of at least α = 0.05. Asterisks are used to define a difference of statistical significance between the FI-RSV vaccinated group and each control group.

## Results

### Development of VERD Following RSV Challenge of FI-RSV-Immunized Cotton Rats

We first established the FI-RSV VERD cotton rat model based on previous reports (25). To this end, we immunized cotton rats twice with FI-RSV followed by challenging the animals with RSV. The controls included: “mock” controls (FI-Mock) representing cell substrate inactivated by formalin, “RSV” controls denoting intranasal immunization with RSV virus (RSV), and “PBS” controls designating PBS buffer immunization. These controls received the same immunization and challenge schedules as the FI-RSV group.

**Figure 1:**
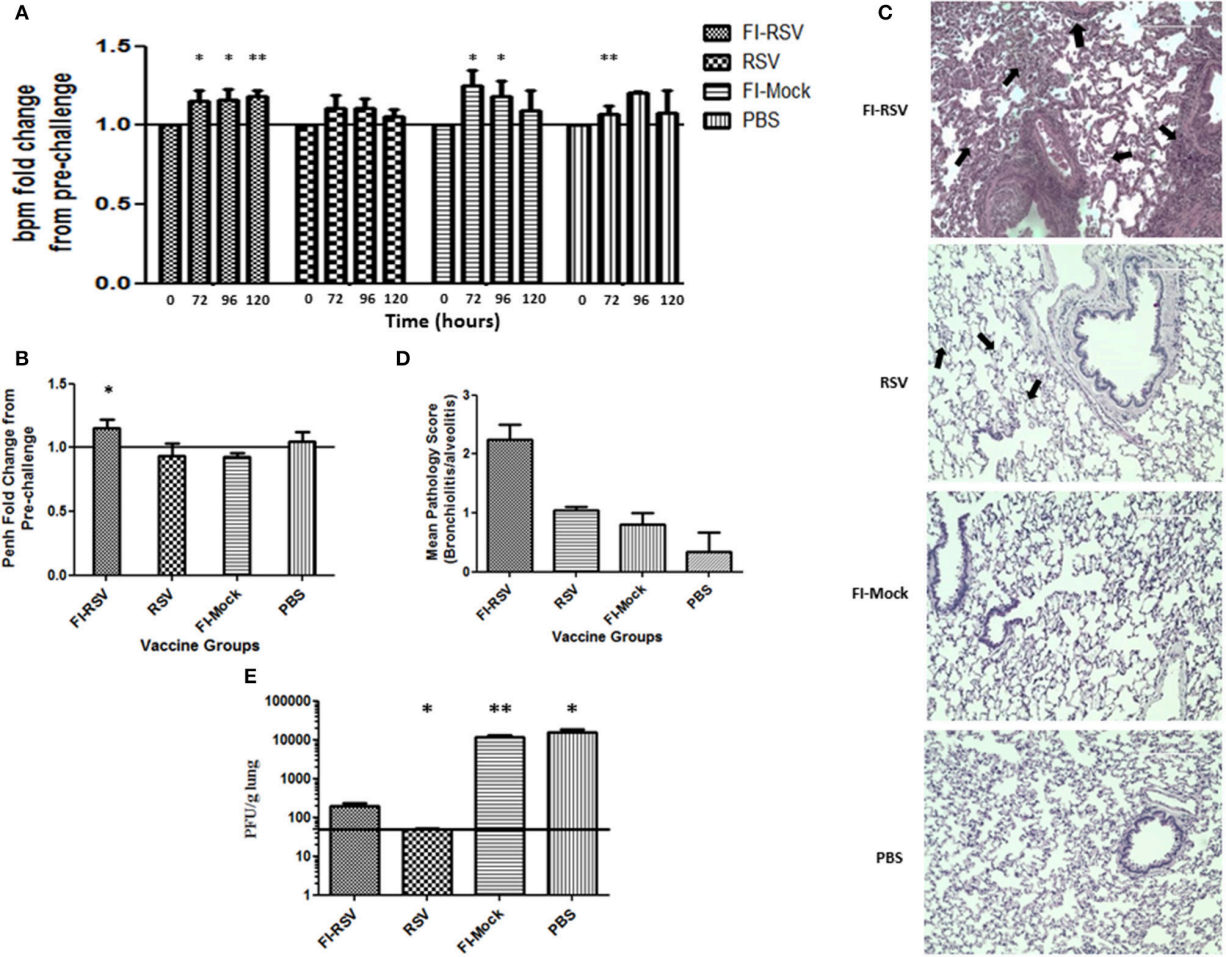
Development of VERD following RSV challenge of FI-RSV-immunized cotton rats. Vaccinated cotton rats were monitored daily for 5 days after RSV challenge for airway obstruction using a whole body plethysmograph (*n* = 6). Results are displayed as fold change from 0 h when challenged with RSV (day 49 of study) for **(A)** breaths per minute (bpm) and **(B)** Penh (120 h after viral challenge). Significant differences between time = 0 h post-challenge for each group and 72, 96, and 120 h of the respective group was assessed using one-way ANOVA, *p < 0.05, **p < 0.01. **(C)** Representative pictures of H&E-stained lung tissue obtained from animals in the indicated groups 5 days after challenge with RSV (40× magnification). **(D)** Lung pathology scores (bronchiolitis and alveolitis) of animals in the indicated groups 5 days after challenge with RSV. (*n* = 6) **(E)** Lung viral titres measured in animals in the indicated groups 5 days after challenge with RSV. Data are represented as mean ± SEM from two independent experiments (*n* = 6). Significant differences between FI-RSV vaccinated group and the other groups were assessed using one-way ANOVA, *p < 0.05, **p < 0.01.

As whole body plethysmography (WBP) is useful in evaluating baseline respiratory patterns that correlate with pulmonary function (23, 34, 37), we used unrestrained WBP to analyse pulmonary function following RSV challenge. Animals were monitored for 5 days post-challenge. FI-RSV-vaccinated cotton rats showed an increased breathing rate (tachypnea) when compared with other control groups. Specifically, tachypnea remained steadily high in the FI-RSV group, whereas stable breathing rate was observed in the RSV group, while only a transient increase in breathing rates was observed in FI-Mock and the PBS groups (Figure 1A).

We also determined airway functional impairment by measuring enhanced pause (Penh), which represents a function of the ratio of peak expiratory flow to peak inspiratory flow and the timing of expiration (represented by the “pause”) (35). As shown in (Figure 1B), a significant increase in Penh was observed on day 5 in animals vaccinated with FI-RSV, whereas no change was found in the other control groups. Collectively, the results from WBP experiments indicate that immunization of these animals with FI-RSV resulted in significant respiratory difficulties in these vaccinated animals upon subsequent RSV challenges.

We next conducted histopathological examination and virus titrations in lung tissues obtained from these animals. Largely in agreement with previous studies (27), we observed prominent alveolitis with infiltrating neutrophils, macrophages and lymphocytes along with a few eosinophils in alveolar spaces in the FI-RSV group, whereas no such pathological observation was made in the other control groups (Figure 1C). Furthermore, peribronchiolitis in the FI-RSV group was characterized with a similar pattern of cellular infiltration as that in alveolitis along with marked perivascular leukocyte infiltrates throughout the lung sections (Figure 1C). These pathological changes were either absent or very mild in other groups. These observations were largely consistent with pathological quantitation, which revealed that the FI-RSV group had higher histopathological scoring than other groups (Figure 1D).

We next evaluated viral burden in the lungs and observed that FI-RSV vaccination markedly reduced viral titers, but that viral clearance was not as complete as it was with RSV immunization. As expected, vaccination with FI-Mock and PBS failed to reduce viral replication in the lungs (Figure 1E). These observations suggest that clinical manifestations, such as tachypnea and airway obstruction, in FI-RSV vaccinated animals are more likely due to exacerbated pulmonary inflammatory reactions than virus-induced pathogenesis (Figure 1A).

**Figure 2:**
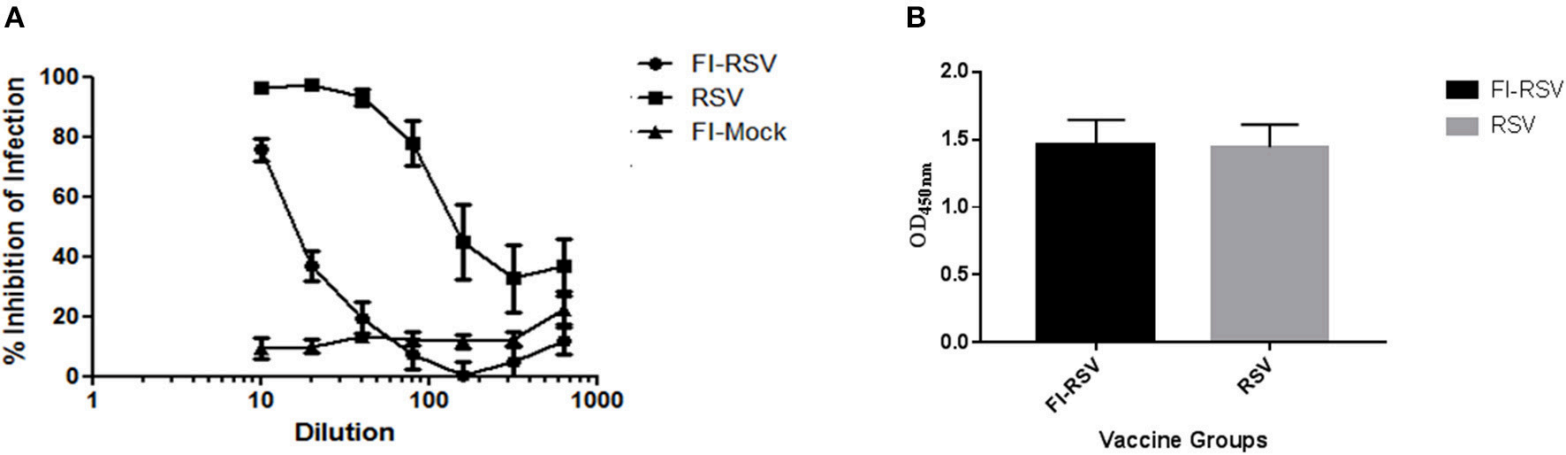
Poor neutralizing antibody profile of cotton rats vaccinated with FI-RSV exhibiting VERD following RSV challenge despite similar levels of IgG. **(A)** Neutralizing antibody response in serum of cotton rats vaccinated with FI-RSV, pre-exposed to RSV or mock vaccinated and challenged with RSV 5 days post-challenge (*n* = 10). **(B)** ELISA quantification of IgG against RSV in the serum of cotton rats 5 days post-challenge in animals that were immunized with FI-RSV or pre-exposed to RSV (*n* = 10).

As the inability of antibodies induced by the FI-RSV vaccine to neutralize the virus is another hallmark of FI-RSV VERD (19, 25), we analyzed neutralizing antibodies induced by FI-RSV. We found that FI-RSV-induced antibodies had a drastically decreased capacity to neutralize the virus (Figure 2A), even though FI-RSV induces similar overall levels of RSV-specific antibodies detected by ELISA compared with immunization with live virus (Figure 2B), in agreement with previous observations (42). Together, the above results confirm the VERD cotton rat model was successfully developed.

### Enrichment and Up-Regulation of Pro-inflammatory Cytokine Signaling in FI-RSV Vaccinated Animals

We next employed quantitative mass spectrometric analysis of the lung tissues. As there is no cotton rat-specific protein database, the mass spectrometric data were first searched against a rat protein database for protein identification and quantification. Further statistical analysis was done on these mass spectrometric datasets to obtain fold changes for FI-RSV, RSV, and FI-Mock over PBS. Significantly altered proteins (*p*-value ≤ 0.05) were evaluated further in IPA (Supplementary Figure 1). Additionally, the raw mass spectrometric data was also searched against a mouse, and a rodent database. When significantly altered proteins generated against these databases were imported into IPA, we observed similar trends in terms of pathway alterations across these databases as was seen with the rat database analysis (data not shown). Thus, we reasoned that the IPA results generated with the rat protein database should be reliable if they are validated with results obtained from immunochemical or qPCR assays using reagents known to be species-specific for cotton rats.

**Figure 3:**
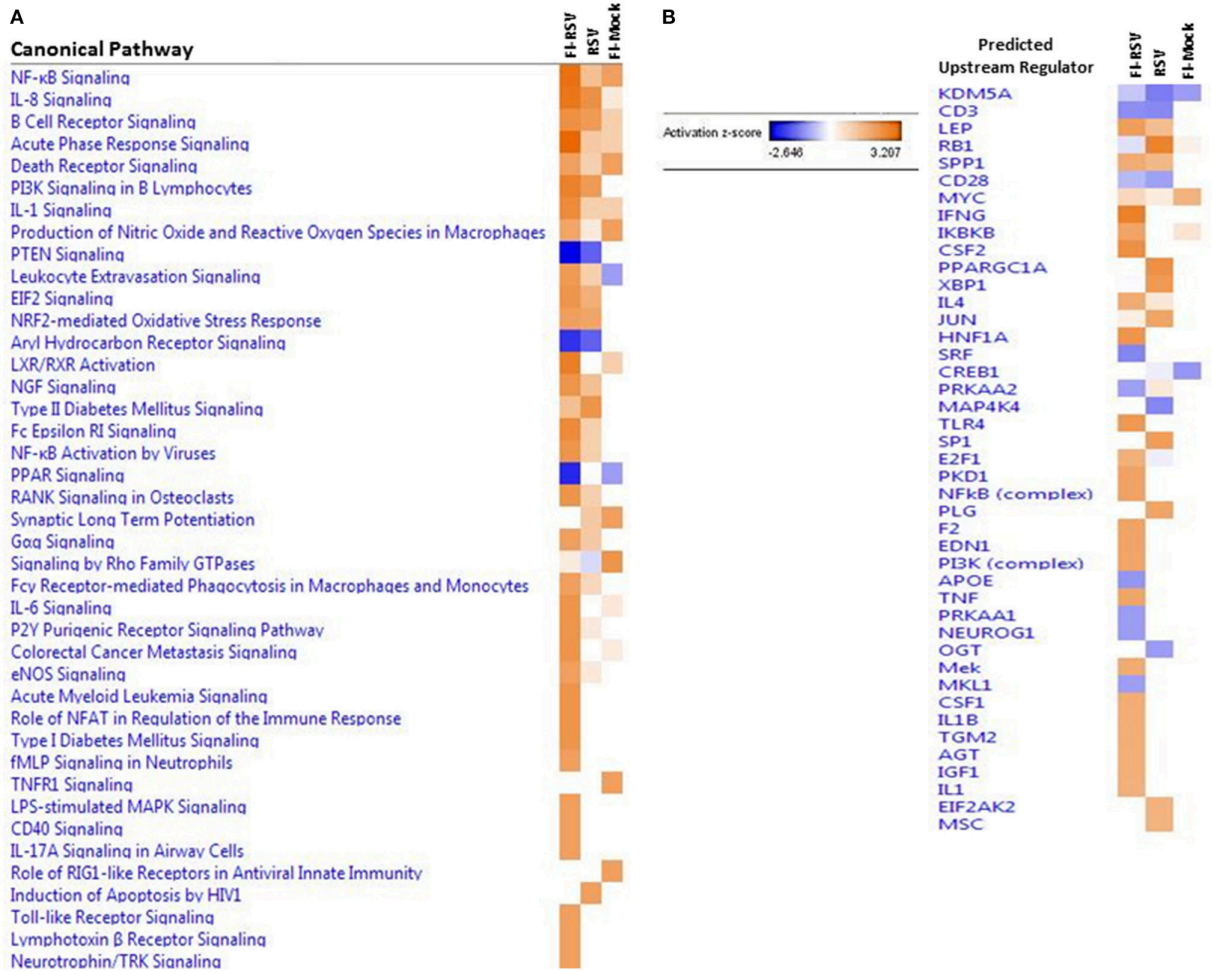
**(A)** Heat map of the comparative analysis of the canonical pathways significantly enriched in the indicated groups relative to PBS controls. **(B)** Heat map of the comparative analysis of the statistically significant upstream regulators predicted to be involved in the indicated groups. For both heat maps, only those with a Z-score equal to ± 2 are displayed. (*n* = 12).

The IPA core analysis of canonical pathways (Figure 3A) revealed a marked pro-inflammatory state in the lungs of FI-RSV animals, which was much less pronounced in the RSV and FI-Mock groups, consistent with the histopathological findings in these animals (Figure 1C). As shown in (Figure 3A), of particular interest in the FI-RSV group was the significant enrichment and upregulation of nuclear factor kappa-light-chain-enhancer of activated B cells (NF-κB) signaling, acute phase response signaling, interleukin 8 (IL-8) signaling (GRO in rodents), interleukin 1 (IL-1) signaling, interleukin 6 (IL-6) signaling, tumor necrosis factor receptor 1 (TNFR1) signaling, interleukin 17A (IL-17A) signaling in airway cells, Fc epsilon RI signaling and PI-3K signaling in B lymphocytes pathways. It is noteworthy that there was increased activation of NF-κB and elevated level of MyD88 (Table 1), suggesting that NF-κB activation might be MyD88-dependent.

**Table 1:** Pathways and proteins associated with lung cellular infiltrates in FI-RSV.

**Cell type**	**Pathway**	**IPA z-score**	**Proteins associated in FI-RSV**
Neutrophil	Cell movement of neutrophils	2.85	ALB(1.341)↑, ALOX5(1.277)↑, ANGPT2(1.34)↑, C2(1.858)↑, C3(1.419)↑, C4A/C4B(1.354)↑, CD151(−1.384)↓, CFB(1.325)↑, CFH(1.969)↑, CHUK(1.427)↑, CORO1A(1.2)↑, CST3(1.244)↑, DOCK5(1.231)↑, F2(1.364)↑, ITGAM(1.419)↑, ITGB2(1.226)↑, ITGB3(1.42)↑, MYD88(1.23)↑, NCF1(1.227)↑, PLG(1.302)↑, PTPN6(1.213)↑, RAC2(1.326)↑, SERPINC1(1.345)↑, SYK(1.222)↑, TSC1(1.269)↑, VTN(1.579)↑.
	Adhesion of neutrophils	2.40	ANGPT2(1.34)↑, C3(1.419)↑, CORO1A(1.2)↑, F2(1.364)↑, FERMT3(1.266)↑, FGA(1.383)↑, ITGAM(1.419)↑, ITGB2(1.226)↑, KNG1(1.332)↑, PTPN6(1.213)↑, VTN(1.579)↑
	Activation of neutrophils	1.76	AHR(−1.22) ↓, C3(1.419)↑, CFH(1.969)↑, CPB2(1.46)↑, CST3(1.244)↑, EPX(1.4)↑, F2(1.364)↑, ITGAM(1.419)↑, ITGB2(1.226)↑, MYD88(1.23)↑
	Binding of neutrophils	1.73	C3(1.419)↑, F2(1.364)↑, FERMT3(1.266)↑, ITGAM(1.419)↑, ITGB2(1.226)↑, PLG(1.302)↑, PTPN6(1.213)↑
	Chemotaxis of neutrophils	1.64	ALOX5(1.277)↑, C3(1.419)↑, CD151(−1.384)↓, CHUK(1.427)↑, COPO1A(1.2)↑, CST3(1.244)↑, DOCK5(1.231)↑, ITGAM(1.419)↑, ITGB2(1.226)↑, PTPN6(1.213)↑, RAC2(1.326)↑, SYK(1.222)↑, TSC1(1.269)↑, VTN(1.579)↑
	IL-8 signaling	2.89	AKT1(1.21)↑, ANGPT2(1.34)↑, BRAF(1.615)↑, CHUK(1.427)↑, GNB2(−1.3)↓, IRAK4(1.321)↑, ITGAM(1.419)↑, ITGB2(1.226)↑, ITGB3(1.42)↑, MAP2K2(1.202)↑, MYL9(−1.338) ↓, NCF2(1.248)↑, RAC2(1.326)↑
	fMLP signaling in neutrophils	2	GNB2(−1.3) ↓, MAP2K2(1.202)↑, NCF1(1.227)↑, NCF2(1.248)↑, NFKB2(1.792)↑
	IL-17A signaling in airway cells	2	AKT1(1.21)↑, CHUK(1.427)↑, MAP2K2(1.202)↑, NFKB2(1.792)↑
Macrophage	Activation of macrophages	2.09	ABHD12(1.487)↑, AHR(−1.22) ↓, ANGPT2(1.34)↑, C3(1.419)↑, F2(1.364)↑, GC(1.337)↑, KNG1(1.332)↑, MAP2K2(1.202)↑, MAPT(−1.3) ↓, MYD88(1.23)↑, PLG(1.302)↑, PTPN6(1.213)↑, SYK(1.222)↑, THBS1(1.281)↑, VTN(1.579)↑
	Chemotaxis of macrophages	2	CHUK(1.427)↑, F2(1.364)↑, ITGB3(1.42)↑, MYD88(1.23)↑, REL(1.788)↑, RPL13A(1.284)↑, THBS1(1.281)↑
	Recruitment of macrophages	1.82	ANGPT2(1.34)↑, BRAF(1.615) ↑, ITGAM(1.419)↑, ITGB2(1.226)↑, MYD88(1.23)↑, NFIC(1.39)↑, PLG(1.302)↑, THBS1(1.281)↑
	Fcy receptor-mediated phagocytosis in macrophages	2	AKT1(1.21)↑, NCF1(1.227)↑, RAC2(1.326)↑, SYK(1.222)↑
Lymphocyte	Quantity of B lymphocytes	2.16	AHR(−1.22) ↓, AKT1(1.21)↑, ARHGDIB(1.31)↑, C3(1.419)↑, CHUK(1.427)↑, ITGB2(1.226)↑, PKN1(1.202)↑, PTPN6(1.213)↑, RAC2(1.326)↑, REL(1.788)↑, SLC39A10(1.661)↑, SYK(1.222)↑, TSC1(1.269)↑
	IL-6 signaling	2.24	AKT1(1.21)↑, CHUK(1.427)↑, MAP2K2(1.202)↑, MAP2K6(1.373)↑, NFKB2(1.792)↑
	IL-1 Signaling	2.45	CHUK(1.427)↑, GNB2(−1.3) ↓, IRAK4(1.321)↑, MAP2K6(1.373)↑, MYD88(1.23)↑, NFKB2(1.792)↑, PRAKACB(1.212)↑
	IL-17A Signaling in airway cells	2	AKT1(1.21)↑, CHUK(1.427)↑, MAP2K2(1.202)↑, NFKB2(1.792)↑
	B cell receptor signaling	2.33	AKT1(1.21)↑, CHUK(1.427)↑, MAP2K2(1.202)↑, MAP2K6(1.373)↑, NFKB2(1.792)↑, PDPK1(1.697)↑, PTPN6(1.213)↑, RAC2(1.326)↑, SYK(1.222)↑
Eosinophil	no specific pathway	N/A	Epx(1.4)↑

*IPA canonical pathways or functional annotations associated with cellular infiltrates observed in the lungs of FI-RSV infected animals. The z-score cut-off was lowered to >1.5 and < -1.5 to reveal those pathways or functional annotations that were not significant yet informative in understating the overall cellular infiltration. Those with z-score >2 and < -2 were considered significant*.

We also observed markedly upregulated oxidative stress canonical pathways. Specifically, nitric oxide and reactive oxygen species (ROS) and nuclear factor erythroid 2 related factor 2 (NRF2)-mediated oxidative stress response pathways were upregulated in FI-RSV, while only one pathway was altered, but to a lesser degree, in the other two control groups, i.e., nitric oxide and ROS in the FI-Mock group or nuclear factor erythroid 2 related factor 2 (NRF2)-mediated oxidative stress response in the RSV group.

To gain better insight into what proteins may be influencing our data set, we employed IPA to predict the upstream regulators of the canonical pathways in FI-RSV. As shown in Figure 3B, upstream regulators including interferon-gamma (IFN-γ), interleukin 4 (IL-4), IL-1, IL1β, were predicted to be activated in the FI-RSV group, largely in agreement with previous studies conducted in mouse, cotton rat and humans (23, 43, 44). It is of note that our analysis also revealed predicted activation of NF-κB complex along with inhibitor of nuclear factor kappa B kinase subunit beta (IKBKB) (Figure 3B), which work in concert in activating the NF-κB signaling pathway (45), in the animals vaccinated with FI-RSV.

**Figure 4:**
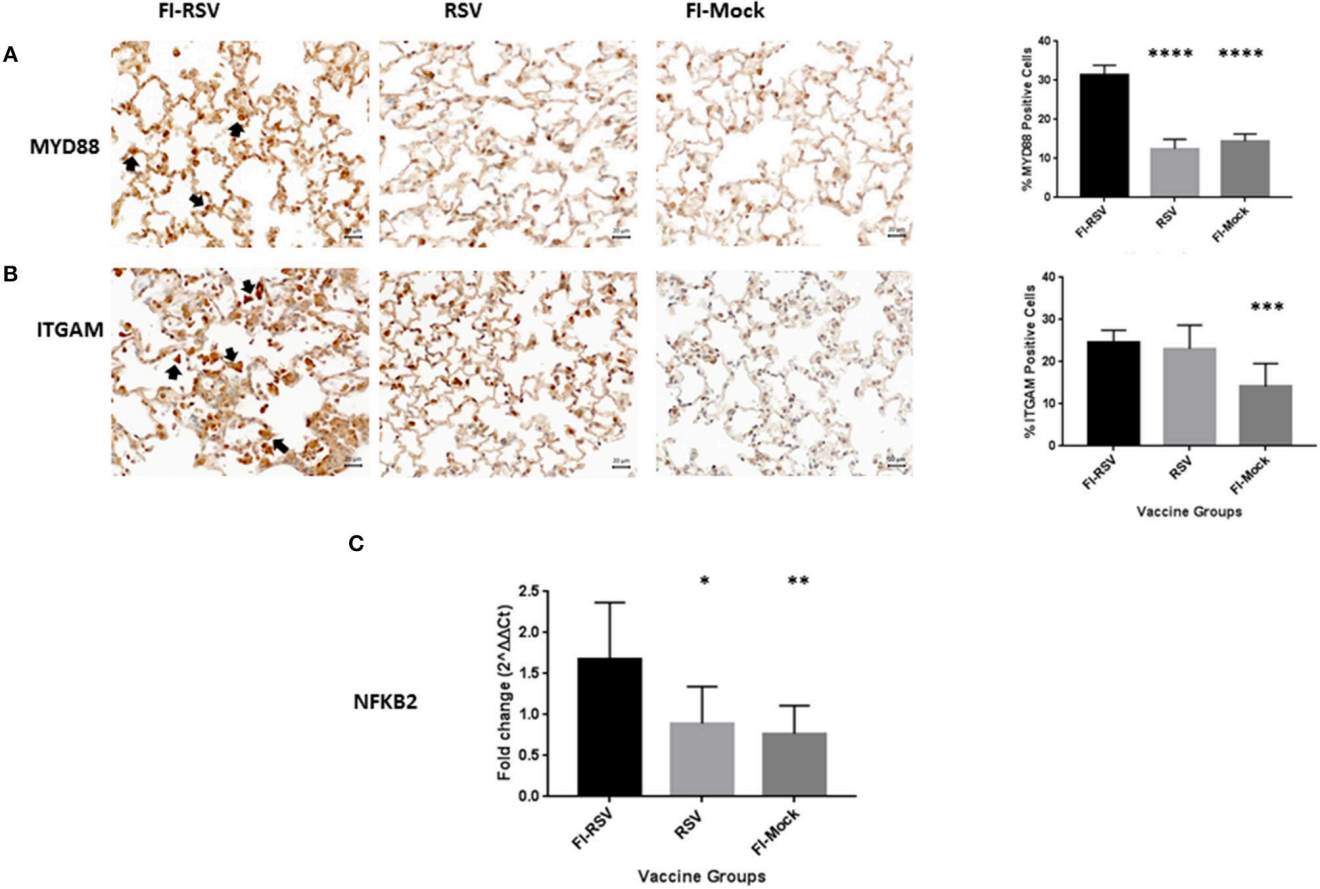
Validation of key proteins in the proteomics datasets. Immunohistochemical detection of MYD88 **(A)** and ITGAM **(B)** in lungs. For each section 15 fields of view were counted for positively stained cells in the alveoli (40× magnification). Black arrows indicate the positive staining by diaminobenzidine (DAB) substrate. The percentages of total area stained as well as the percentages of positive cells for each cytokine were calculated using one-way ANOVA, *p < 0.05, **p < 0.01, ***p < 0.001 (*n* = 6). Gene expression of NFKB2 **(C)** in the lungs was normalized to β-actin and fold change was expressed over the PBS group using the ΔΔCt method. Statistical differences in fold change were calculated using one-way ANOVA, *p < 0.05, **p < 0.01, ***p < 0.001, ****p < 0.0001 (n = 6).

To validate these findings we more closely evaluated key proteins in our datasets using immunohistochemistry and qPCR methodology. As revealed from the proteomics findings, we observed a marked increase in MYD88 in the FI-RSV group (Figure 4A) which is well-studied and known to play a key role in inflammatory pathways (46). Also in agreement with the proteomics data, we observed an increase in ITGAM in both the FI-RSV and RSV groups, suggesting that the upregulation of this protein is antigen driven [B]. We were unable to find a suitable commercial antibody for the detection of NFκB2; however, we were able to determine the gene expression levels of this marker by qPCR (Figure 4C). We found that the gene expression levels of NFκB2 were highest in the FI-RSV group compared to the RSV and FI-Mock groups.

### Higher Levels of IL-1 and IL-6 Cytokines and GRO Chemoattractant in FI-RSV Vaccinated Animals

**Figure 5:**
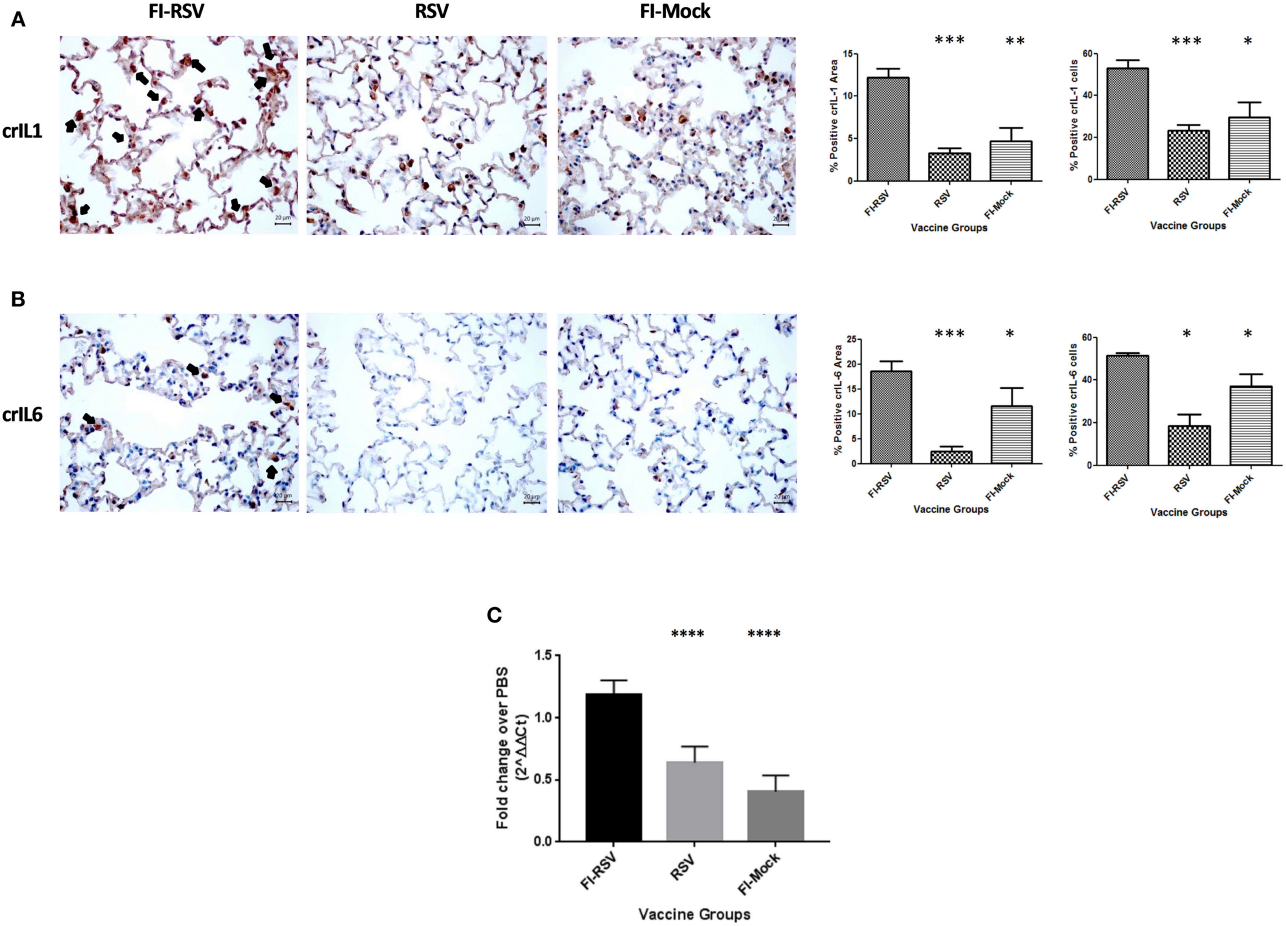
Higher expression levels of IL-1 and IL-6 cytokines and chemoattractant GRO in FI-RSV vaccinated cotton rats. Detection of pulmonary interleukin-1 **(A)** and interleukin-6 **(B)** was conducted using immunohistochemistry. For each section 15 fields of view were counted for positively stained cells in the alveoli (40× magnification). Black arrows indicate the positive staining by diaminobenzidine (DAB) substrate. The percentages of total area stained as well as the percentages of positive cells for each cytokine were calculated using one-way ANOVA, *p < 0.05, **p < 0.01, ***p < 0.001 (*n* = 6). Gene expression of GRO **(C)** in the lungs was normalized to β-actin and fold change was expressed over the PBS group using the ΔΔCt method. Statistical differences in fold change were calculated using one-way ANOVA, *p < 0.05, **p < 0.01, ***p < 0.001, ****p < 0.0001 (n = 6).

To validate some critical canonical pathways elucidated by IPA, we conducted immunohistochemical staining or qPCR analysis of the lungs derived from the animals using available cotton rat species-specific reagents. As the IPA data showed an increased z-score for the IL-1, IL-6, and IL-8 signaling cascades in the VERD animals, we sought to further evaluate these key cytokines and chemokine. We used antibodies against cotton rat IL-1 and IL-6, as well as gene specific primers against cotton rat GRO (the IL-8 related protein in rodents) to determine if these cytokines were elevated in pulmonary tissues of VERD animals. As shown in (Figures 5A,B), we observed substantially increased levels of both the IL-1 and IL-6 cytokines 5 days post-challenge in the lungs of the FI-RSV group relative to RSV or FI-Mock groups. Notably, the higher levels of both cytokines were observed not only in the number of cells producing IL-1 or IL-6 cytokines, but also in the overall area where soluble IL-1 or IL-6 was present. The gene expression of GRO was also increased in the FI-RSV group compared to the RSV and FI-Mock groups (Figure 5C), consistent with the findings of others (17).

### IPA Downstream Effects Analysis Predicts Possible Hematological Disorder in VERD Animals

**Figure 6:**
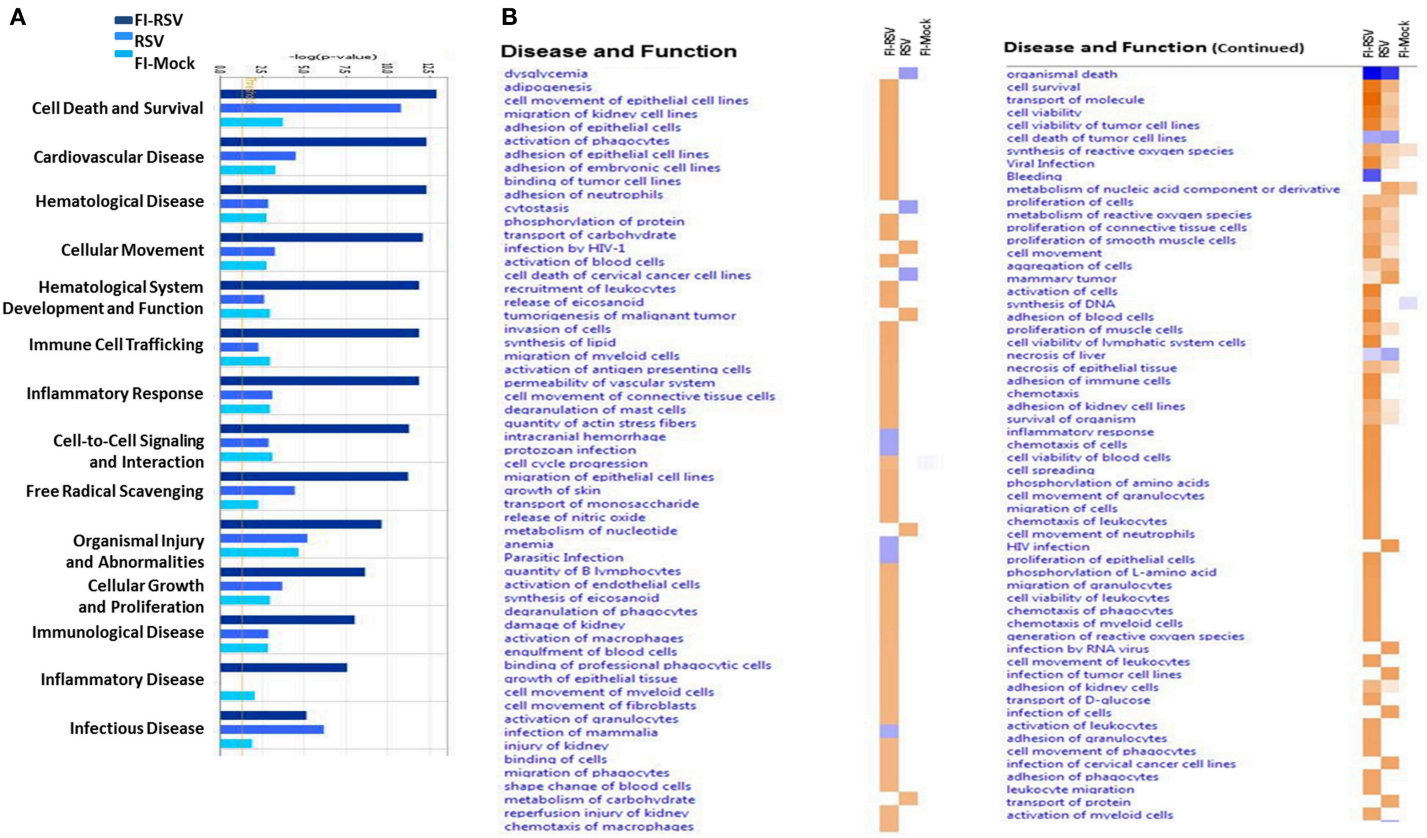
Predicted disease and functional outcomes from Ingenuity Pathway Analysis highlights the possibility of a hypercoagulation state. IPA downstream analysis results are displayed with the *p*-value of overlap **(A)** showing the likelihood that the disease and/or functional outcome occur. Those that gave a –log1.3 were considered significant (yellow line). Only the first 14 most highly predicted outcomes are displayed. **(B)** Heat map of the z-score (>2 or <-2) showing the degree of up-regulation or down regulation of the respective disease or functional annotations in each of the groups (*n* = 12).

We next utilized IPA to conduct downstream effects analyses to determine if the observed proteomic profiles predict alterations in biological functions or diseases. The analysis predicted potential impacts on cellular movement, immune cell trafficking, inflammatory response, immunological disease, inflammatory disease, cardiovascular disease, and hematological diseases (Figure 6). Consistent with histopathological and immunochemical examination of the pulmonary tissues (Figures 1,3), significantly altered proteins in FI-RSV VERD animals were associated with increased levels of inflammatory response, immune cell trafficking, cellular movement, immunological responses (Figure 6A).

Markedly increased cell trafficking was also noted in FI-RSV group. Expressly, we observed pathways related specifically to neutrophilic infiltration, i.e., cell movement of neutrophils and adhesion of neutrophils (Figure 6B, Table 1), exclusively in the FI-RSV group. Activation of macrophages and chemotaxis of macrophages (Figure 6B) (Table 1) were also unique to this treatment group. In relation to general leukocyte trafficking, proteins associated with chemotaxis of leukocytes, chemotaxis of phagocytes, cell movement of leukocytes, activation of leukocytes, and recruitment of leukocytes were also over-represented, with these protein sets exhibiting high (>2) *z*-scores (up-regulated) exclusively in FI-RSV (Figure 6B).

Finally, we observed a significant increase in functional annotations related to oxidative stress. These include synthesis, metabolism of, and generation of ROS, and release of nitric oxide, all of which gave higher (>2) *z*-scores in the FI-RSV group (Figure 6B).

### Increased Fibrinogen and Fibrin Accumulation in Pulmonary Tissues of FI-RSV Vaccinated Animals

Two pathways identified in the downstream effects analysis were associated with hematological disorder in FI-RSV VERD animals (Figure 6A). It is of note that in the FI-RSV group, the bleeding pathway (Supplementary Tables 1, 2) and pathways associated with hemorrhage and anemia showed marked down-regulation (Figure 6B, Supplementary Table 2). Moreover, the adhesion of blood cells and activation of blood cells pathways were significantly up-regulated in the FI-RSV group (Figure 6B, Supplementary Table 2). These pathways, along with up-regulation of the cell viability of red blood cells pathway, the engulfment of blood cells pathway, and shape change of blood cells (Figure 6B, Supplementary Table 2), suggest a homeostatic imbalance between coagulation and fibrinolysis (47). Moreover, the disease and functional annotations analysis revealed multiple pathways related to blood clotting (Supplementary Table 3).

The coagulation cascade is well-studied, with its activation resulting in the formation of fibrin clots derived from the precursor, fibrinogen. By examining pulmonary proteomic profiles derived from FI-RSV VERD animals, we noted that the levels of some important proteins in this cascade were increased in FI RSV VERD animals but not in RSV or the mock controls. Specifically, coagulation factors II (thrombin) and XI were increased in the FI-RSV group, while another key protein, fibrinogen alpha chain, was also up-regulated (Supplementary Table 1).

**Figure 7:**
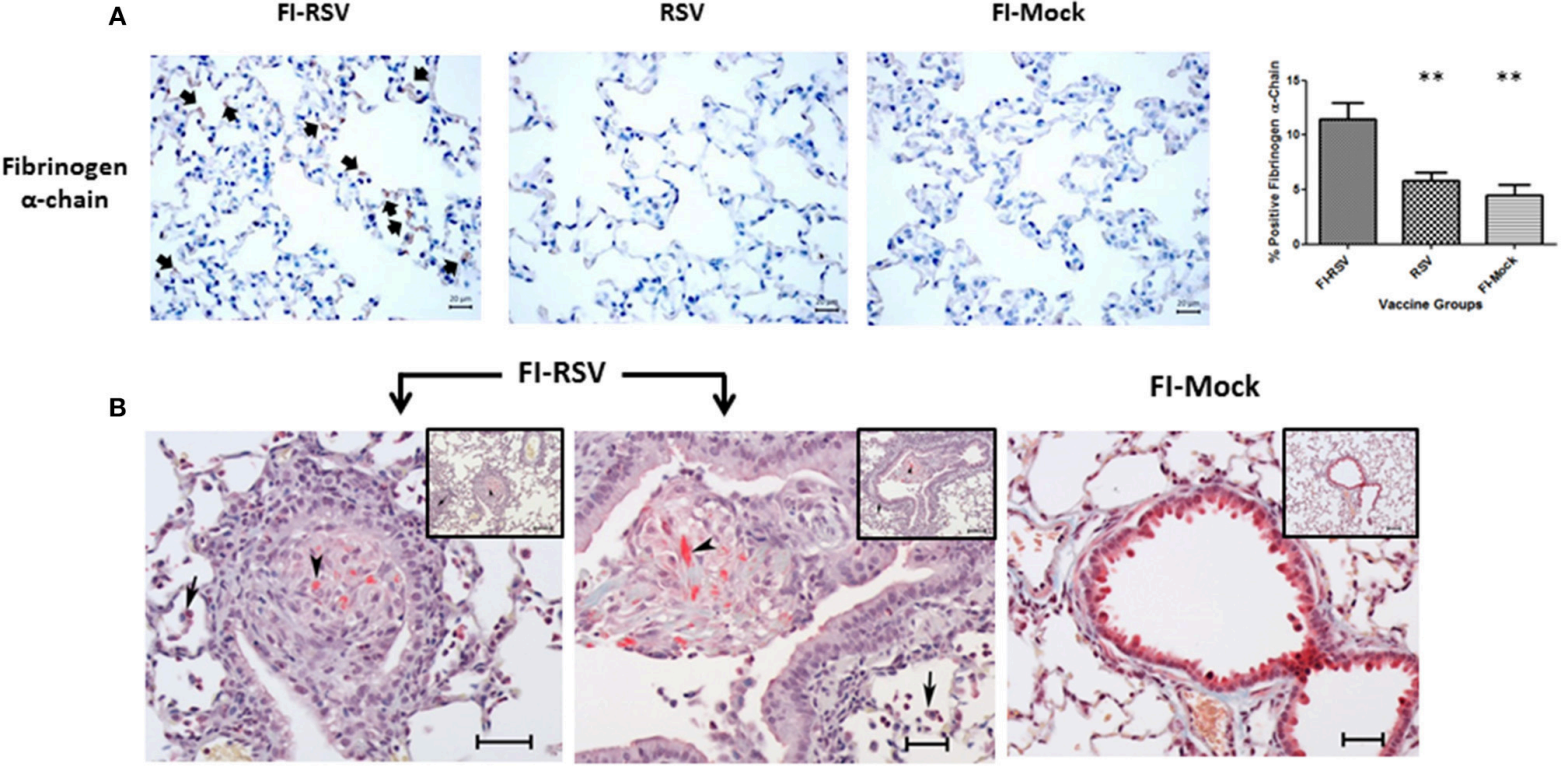
FI-RSV vaccinated cotton rats demonstrate increased expression of fibrinogen alpha-chain and the presence of fibrin deposits 5 days after RSV challenge. **(A)** Immunohistochemical detection of fibrinogen alpha-chain in the lung. Pictures show a representative staining of each vaccine group at 40× objective. The percentage of total area stained was calculated using one-way ANOVA, **p < 0.01, (*n* = 6). **(B)** MSB staining for fibrin. Pictures show a representative area of lung containing positive stain for fibrin (arrowhead). In the FI-RSV group, alveolitis with a mixture of macrophages and neutrophils in alveolar spaces (full arrows) was observed. Additionally, there is bronchiolitis with mainly macrophages and some neutrophils and small amounts of red staining (MSB stain) fibrin (arrowhead) in the lumen in this vaccine group. Inset scale = 120 μm, large picture scale = 50 μm.

Fibrinogen is a protein of particular interest because it is a key player in the formation of fibrin clots in the coagulation cascade and an important acute phase response protein. Increased levels of fibrinogen have been associated with increased risk of venous thromboembolism (48). Therefore, we measured the levels of fibrinogen alpha-chain using immunohistochemistry and found a markedly increased level of fibrinogen in the alveolar areas of lungs derived from FI-RSV immunized animals (Figure 7A). Moreover, when lungs of these animals were stained for deposits of insoluble fibrin by MSB staining, exuded extracellular fibrin deposits were found in the bronchiolar lumen of the lung tissues derived from the FI-RSV vaccinated animals (Figure 7B). Additionally, there were discernible increases in the alveolar septa displaying hyperaemic and slugging of red cells. The pathological examinations of the lung tissues were conducted independently by two veterinary pathologists in a blind fashion. Taken together, these results collectively suggest that FI-RSV vaccination in conjunction with subsequent viral infection could result in pulmonary hypercoagulation.

### Impaired Fibrinolysis in FI-RSV VERD Animals

**Figure 8:**
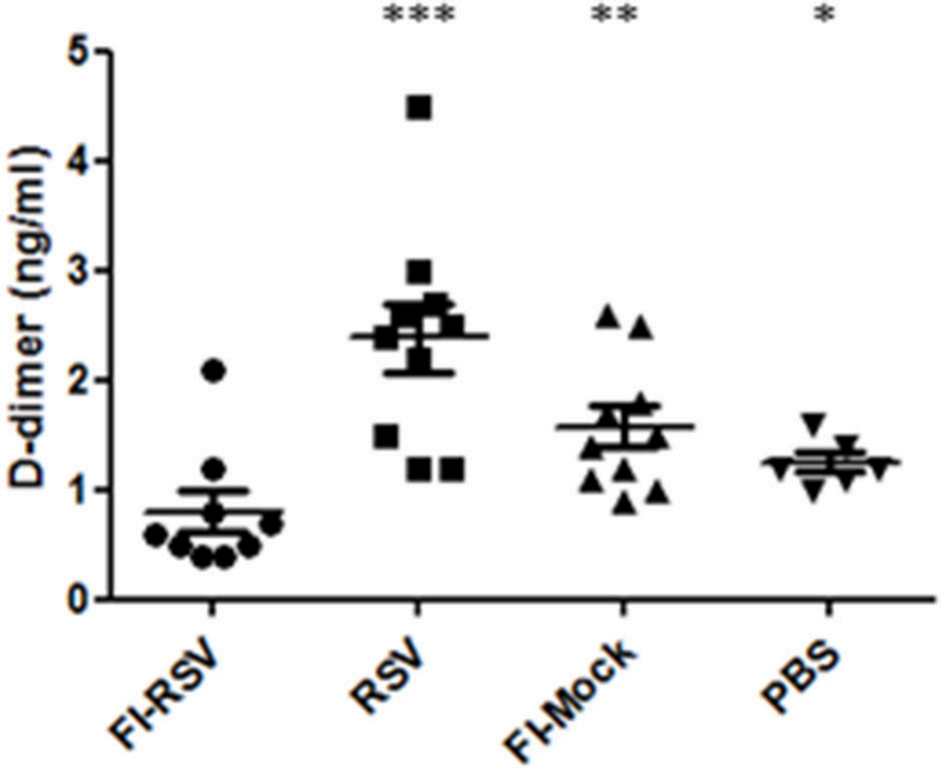
FI-RSV vaccinated cotton rats show low plasma D-dimer levels 5 days after RSV challenge. Plasma D-dimer levels were measure with ELISA. Significant differences between FI-RSV and the other vaccine groups were calculated using one-way ANOVA, *p < 0.05, **p < 0.01, ***p < 0.001 (*n* = 10).

As we observed increased deposition of fibrin in the pulmonary tissues in FI-RSV vaccinated animals, we next determined whether any impairment in fibrinolysis was present in the diseased animals. It is well-known that upon vascular injury, the dynamic hematological system balances blood coagulation and fibrinolysis to ensure a proper wound-healing response. We analyzed the amounts of D-dimer, a fibrin degradation product, in the plasma from FI-RSV and control groups and found a marked decrease in D-dimer levels in FI-RSV vaccinated animals as compared to the other control groups (Figure 8), suggesting a deficiency in fibrinolysis. Furthermore, D-dimer levels were highest in the RSV group, suggesting that the homeostatic balance between coagulation and fibrinolysis was functioning normally. Not surprisingly, following vaccination with no RSV proteins, i.e., FI-Mock or PBS, the D-dimer levels were lower than RSV vaccination. The tilt in the homeostatic balance toward hemostasis and lower levels of fibrinolysis in these animals could be explained in part by the high viral burden in these animals (49). Importantly however, the D-dimer levels were lowest in the FI-RSV group despite the absence of active viral replication compared with all other groups, suggesting the likelihood of impediment in fibrinolysis.

## Discussion

RSV is a major cause of viral lower respiratory tract infections among seniors, infants and young children. Despite several decades in vaccine research and development, no vaccine is available. While tremendous efforts have been continuously put into developing RSV vaccine, the mechanisms underlying RSV vaccine associated VERD remains to be fully understood.

Several lines of evidences prompted us to carry out the current study. Firstly, VERD is not exclusive to formalin inactivated vaccine as other forms of RSV vaccines have been reported to cause VERD (9, 12–15), revealing the complexity of the molecular mechanisms which is unlikely to restricted to the use of inactivated agent/method. Secondly, while significant progress has been made in recent years toward better understanding VERD, most of the studies involved investigation of specific pathways in mice; thus, our understanding of the disease would be advanced by employing a holistic approach to obtain a global profile of the host responses to VERD. Thirdly, while a quantitative proteomics approach has been used to investigate VERD in mice (9), with the identification of biomarkers indicative of eosinophil and neutrophil influx in mice, it would be of merit to investigate the mechanism in the cotton rat model, given its closer resemblance to humans in terms of VERD pathology.

The challenge associated with the cotton rat model is that the genome of this species remains to be sequenced, with limited research reagents available for further verifications of the proteins using other assays such as immunoassay and qPCR. However, in our study when the raw mass spectrometric data was searched against a mouse, rat and a rodent database, we observed similar trends in terms of pathway alterations across these databases (data not shown). Therefore, we concluded that the quantitative proteomics results generated with the rat protein database were reliable since key components were later validated with other assays using reagents cross-reactive with proteins or mRNA of cotton rats (Figures 4, 5, 7). Using this unbiased quantitative proteomics, we observed VERD involved in a wide range of host responses leading to severe pulmonary lesions, resulting in significant clinical manifestations in cotton rats. While exacerbated pulmonary inflammation are in agreement with previous observations, our findings reported here are novel in that they either connect various functional pathways to pathological presentation/clinical manifestation, or represent new pathways which have not been reported before in association with VERD.

It is of note that some individual cytokine expression at the mRNA level were examined by others (17), we observed the marked upregulation of IL-1, IL-6, IL-8, and IL-17A signaling pathways which may be mediated through NF-κB and MYD88 (Figure 4) signaling transduction as both components have been upregulated. Moreover, it is worth noting that IL-1, IL-8, and IL17A all play important roles in smooth muscle contraction and contribute to the impaired elasticity of the pulmonary tissues through bronchio/alveolar constriction (50, 51), likely resulting in the increased tachypnea and airway obstruction (Figures 1A,B).

Our observed up-regulation of the Fc epsilon RI pathway in the FI-RSV group is also interesting, given its involvement in allergic reaction or hyperreactivity by facilitating the secretion of allergic mediators (52). Blocking this pathway was found to make the mouse resistant to RSV re-infection (53). Here, our findings suggest that this pathway might also be involved in VERD development.

Our study also revealed insight into the mechanisms underlying the cellular infiltration observed in pulmonary tissues. Specifically, the increased levels of IL-8 and IL-17A could be associated with neutrophil recruitment while IL-1, IL-6 and IL-17A were likely the main reasons for lymphocyte influx (Table 1; Figure 3). Furthermore, the elevated levels of ITGAM (Figure 4) and ITGB2 may contribute to macrophage adhesion (Table 1, Figure 6B) and both have been observed by others to be increased in a mouse model of VERD using a vaccinia virus vectored vaccine (9). Interestingly, we also observed increased levels of eosinophil peroxidase (Epx) as that found in the mouse proteomics study (9), but the infiltration of eosinophils was not as massive as that of neutrophils, macrophages, or lymphocytes.

For the first time, we observed homeostatic imbalance between hemostasis and fibrinolysis associated with VERD. Specifically, we observed increased levels of key components promoting coagulation including factor IX, factor II, and fibrinogen (Table 1, Supplementary Table 1). These observations were confirmed by quantitative detection of fibrinogen and identification of increased fibrin deposits by MSB staining of the pulmonary tissues. Moreover, the observed lower levels of D-dimer in the plasma samples in the FI-RSV vaccinated groups compared with the control groups revealed an impediment in fibrinolysis, which lends support to the findings of the increased pulmonary fibrinogen/fibrin depositions (Figures 7, 8). These pathological and immunochemical findings have not been reported in the past studies, likely due to the tissues being stained with haematoxylin and eosin (H/E) whereas in addition to H/E staining we also employed more specific and sensitive assays including immunostaining and MSB staining for fibrinogen and fibrin.

Our study also helps elucidating the mechanism underlying homeostatic imbalance between coagulation and fibrinolysis in VERD. The homeostatic imbalance is likely facilitated by increased levels of IL-1β, IL-6, IL-8, and neutrophil infiltration in the lungs, given that these cytokines have been implicated in enhancing coagulation. Specifically, IL-6 up-regulates tissue factor, while IL-1/IL-1β enhances production of plasminogen activator inhibitor-1 and downregulates thrombomodulin (54). Furthermore, IL-8 triggers platelet activation, while neutrophils promote fibrin formation (55, 56). Moreover, it is known that anticoagulants could abolish IL-1, IL-6, and IL-8 production through inhibition of NFκB activation and platelet aggregation (57). Taken together, it would be interesting to explore future treatment strategies by restoring homeostatic imbalance between coagulation and fibrinolysis, in addition to targeted suppression of excessive inflammatory reactions, while it could be beneficial to employ this approach to investigate VERD induced by other forms of vaccines. Nevertheless, given the significantly decreased level of plasma D-dimer, it would be advisable to determine hematological biomarkers for RSV vaccine evaluation.

In summary, we employed an integrated systems biology approach to investigate RSV vaccine associated respiratory diseases in cotton rats. We unraveled complex but interdependent host responses in the development of vaccine-associated diseases; our work illuminates the molecular mechanisms underlying enhanced respiratory diseases and provides potentially critical information to facilitate the development and evaluation of safe and effective vaccines against RSV infection.

## Data Availability

Datasets are available on request.

## Author Contributions

MR, MC, AM, WC, LL, JL, JC, and TC performed the experiments. JG performed the statistical analysis. WC, AF, MR-M, AH, CY, CL, JC, GV, TC, AF, and XL conceived the project. All authors contributed to manuscript preparation.

### Conflict of Interest Statement

The authors declare that the research was conducted in the absence of any commercial or financial relationships that could be construed as a potential conflict of interest.
