# Supplementary material for: Unveiling Integrated Functional Pathways Leading to Enhanced Respiratory Disease Associated With Inactivated Respiratory Syncytial Viral Vaccine
Source: Front Immunol. 2019 Mar 29;10:597. doi: 10.3389/fimmu.2019.00597 (PMC6449435; doi:10.3389/fimmu.2019.00597)
Supplement: Supplementary file 1 [file Table_1.docx]

Supplementary Table1: Bleeding pathway proteins.

| **Symbol** | **Entrez Gene Name** | **FI-RSV** | **RSV** | **FI-Mock** |
| --- | --- | --- | --- | --- |
| ALB | albumin | 1.341 | NF | NF |
| ALOX5 | arachidonate 5-lipoxygenase | 1.277 | NF | NF |
| ANGPT2 | angiopoietin 2 | 1.34 | NF | 1.497 |
| BIRC6 | baculoviral IAP repeat containing 6 | 1.306 | NF | NF |
| BRAF | B-Raf proto-oncogene, serine/threonine kinase | 1.615 | 1.869 | 1.703 |
| C3 | complement component 3 | 1.419 | NF | NF |
| C4A/C4B | complement component 4B (Chido blood group) | 1.354 | NF | NF |
| CDK2 | cyclin dependent kinase 2 | 1.329 | NF | NF |
| CDK4 | cyclin dependent kinase 4 | 1.349 | 1.37 | NF |
| CST3 | cystatin C | 1.244 | 1.216 | 1.499 |
| F2 | coagulation factor II, thrombin | 1.364 | NF | NF |
| F9 | coagulation factor IX | 1.568 | NF | NF |
| FERMT3 | fermitin family member 3 | 1.266 | 1.171 | NF |
| FGA | fibrinogen alpha chain | 1.383 | NF | NF |
| INSR | insulin receptor | 1.428 | NF | NF |
| ITGB3 | integrin subunit beta 3 | 1.42 | NF | NF |
| MYD88 | myeloid differentiation primary response 88 | 1.23 | NF | NF |
| PDPK1 | 3-phosphoinositide dependent protein kinase 1 | 1.697 | 1.785 | NF |
| PLG | plasminogen | 1.302 | 1.342 | NF |
| RASA1 | RAS p21 protein activator 1 | 1.343 | 1.313 | NF |
| REL | REL proto-oncogene, NF-kB subunit | 1.788 | 1.937 | 1.932 |
| SERPINC1 | serpin family C member 1 | 1.345 | NF | NF |
| SYK | spleen associated tyrosine kinase | 1.222 | NF | NF |
| THBS1 | thrombospondin 1 | 1.281 | 1.146 | NF |

Fold change of proteins found using quantitative proteomics analysis of the lungs of cotton rats vaccinated with FI-RSV, RSV, or FI-Mock. This pathway was given a z-score of -4.246. NF = protein not found in the dataset. Proteins with fold change >1.2 were considered significant.
