# Supplementary material for: Unveiling Integrated Functional Pathways Leading to Enhanced Respiratory Disease Associated With Inactivated Respiratory Syncytial Viral Vaccine
Source: Front Immunol. 2019 Mar 29;10:597. doi: 10.3389/fimmu.2019.00597 (PMC6449435; doi:10.3389/fimmu.2019.00597)
Supplement: Supplementary file 2 [file Table_2.docx]

Supplementary Table2: z-score values corresponding to Figure 5B.

| **Diseases and Biological Functions** | **FI-RSV** | **RSV** | **FI-Mock** |
| --- | --- | --- | --- |
| organismal death | -6.41 | -5.02 | NF |
| cell survival | 3.86 | 2.09 | NF |
| transport of molecule | 4.28 | 1.48 | NF |
| cell viability | 3.86 | 1.81 | NF |
| cell viability of tumor cell lines | 3.47 | 1.50 | NF |
| cell death of tumor cell lines | -2.17 | -2.40 | NF |
| synthesis of reactive oxygen species | 2.49 | 1.18 | 0.89 |
| Viral Infection | 3.32 | 0.98 | NF |
| Bleeding | -4.25 | NF | NF |
| metabolism of nucleic acid component or derivative | NF | 2.42 | 1.62 |
| proliferation of cells | 2.00 | 1.99 | NF |
| metabolism of reactive oxygen species | 2.71 | 1.23 | NF |
| proliferation of connective tissue cells | 2.28 | 1.54 | NF |
| proliferation of smooth muscle cells | 2.58 | 1.07 | NF |
| cell movement | 2.87 | 0.63 | NF |
| aggregation of cells | 1.39 | 2.11 | NF |
| mammary tumor | 0.74 | 2.73 | NF |
| activation of cells | 3.45 | NF | NF |
| synthesis of DNA | 2.62 | NF | -0.74 |
| adhesion of blood cells | 3.30 | NF | NF |
| proliferation of muscle cells | 2.42 | 0.86 | NF |
| cell viability of lymphatic system cells | 3.24 | NF | NF |
| necrosis of liver | -1.13 | -2.10 | NF |
| necrosis of epithelial tissue | 2.00 | 1.21 | NF |
| adhesion of immune cells | 3.17 | NF | NF |
| chemotaxis | 3.08 | NF | NF |
| adhesion of kidney cell lines | 2.40 | 0.66 | NF |
| survival of organism | 2.16 | 0.83 | NF |
| inflammatory response | 2.98 | NF | NF |
| chemotaxis of cells | 2.96 | NF | NF |
| cell viability of blood cells | 2.96 | NF | NF |
| cell spreading | 2.93 | NF | NF |
| phosphorylation of amino acids | 2.93 | NF | NF |
| cell movement of granulocytes | 2.90 | NF | NF |
| migration of cells | 2.89 | NF | NF |
| chemotaxis of leukocytes | 2.86 | NF | NF |
| cell movement of neutrophils | 2.85 | NF | NF |
| HIV infection | NF | 2.84 | NF |
| proliferation of epithelial cells | 2.83 | NF | NF |
| phosphorylation of L-amino acid | 2.76 | NF | NF |
| migration of granulocytes | 2.70 | NF | NF |
| cell viability of leukocytes | 2.68 | NF | NF |
| chemotaxis of phagocytes | 2.68 | NF | NF |
| chemotaxis of myeloid cells | 2.67 | NF | NF |
| generation of reactive oxygen species | 2.66 | NF | NF |
| infection by RNA virus | NF | 2.63 | NF |
| cell movement of leukocytes | 2.62 | NF | NF |
| infection of tumor cell lines | NF | 2.61 | NF |
| adhesion of kidney cells | 2.02 | 0.58 | NF |
| transport of D-glucose | 2.57 | NF | NF |
| infection of cells | NF | 2.57 | NF |
| activation of leukocytes | 2.57 | NF | NF |
| adhesion of granulocytes | 2.56 | NF | NF |
| cell movement of phagocytes | 2.55 | NF | NF |
| infection of cervical cancer cell lines | NF | 2.53 | NF |
| adhesion of phagocytes | 2.52 | NF | NF |
| leukocyte migration | 2.50 | NF | NF |
| transport of protein | NF | 2.45 | NF |
| activation of myeloid cells | 2.44 | NF | NF |
| dysglycemia | NF | -2.44 | NF |
| adipogenesis | 2.42 | NF | NF |
| cell movement of epithelial cell lines | 2.42 | NF | NF |
| migration of kidney cell lines | 2.42 | NF | NF |
| adhesion of epithelial cells | 2.40 | NF | NF |
| activation of phagocytes | 2.40 | NF | NF |
| adhesion of embryonic cell lines | 2.40 | NF | NF |
| adhesion of epithelial cell lines | 2.40 | NF | NF |
| binding of tumor cell lines | 2.40 | NF | NF |
| adhesion of neutrophils | 2.39 | NF | NF |
| cytostasis | NF | -2.39 | NF |
| phosphorylation of protein | 2.38 | NF | NF |
| transport of carbohydrate | 2.38 | NF | NF |
| infection by HIV-1 | NF | 2.38 | NF |
| activation of blood cells | 2.37 | NF | NF |
| cell death of cervical cancer cell lines | NF | -2.37 | NF |
| recruitment of leukocytes | 2.36 | NF | NF |
| release of eicosanoid | 2.34 | NF | NF |
| tumorigenesis of malignant tumor | NF | 2.33 | NF |
| invasion of cells | 2.33 | NF | NF |
| synthesis of lipid | 2.32 | NF | NF |
| migration of myeloid cells | 2.31 | NF | NF |
| activation of antigen presenting cells | 2.28 | NF | NF |
| permeability of vascular system | 2.27 | NF | NF |
| cell movement of connective tissue cells | 2.27 | NF | NF |
| degranulation of mast cells | 2.26 | NF | NF |
| quantity of actin stress fibers | 2.23 | NF | NF |
| intracranial hemorrhage | -2.22 | NF | NF |
| protozoan infection | -2.22 | NF | NF |
| cell cycle progression | 2.04 | NF | -0.17 |
| migration of epithelial cell lines | 2.21 | NF | NF |
| growth of skin | 2.20 | NF | NF |
| transport of monosaccharide | 2.19 | NF | NF |
| release of nitric oxide | 2.19 | NF | NF |
| metabolism of nucleotide | NF | 2.19 | NF |
| anemia | -2.18 | NF | NF |
| Parasitic Infection | -2.18 | NF | NF |
| quantity of B lymphocytes | 2.16 | NF | NF |
| activation of endothelial cells | 2.16 | NF | NF |
| synthesis of eicosanoid | 2.14 | NF | NF |
| degranulation of phagocytes | 2.12 | NF | NF |
| damage of kidney | 2.12 | NF | NF |
| activation of macrophages | 2.09 | NF | NF |
| engulfment of blood cells | 2.08 | NF | NF |
| binding of professional phagocytic cells | 2.07 | NF | NF |
| growth of epithelial tissue | 2.07 | NF | NF |
| cell movement of myeloid cells | 2.07 | NF | NF |
| cell movement of fibroblasts | 2.07 | NF | NF |
| activation of granulocytes | 2.07 | NF | NF |
| infection of mammalia | -2.05 | NF | NF |
| injury of kidney | 2.03 | NF | NF |
| binding of cells | 2.02 | NF | NF |
| migration of phagocytes | 2.02 | NF | NF |
| shape change of blood cells | 2.01 | NF | NF |
| metabolism of carbohydrate | NF | 2.01 | NF |
| reperfusion injury of kidney | 2.00 | NF | NF |
| chemotaxis of macrophages | 2.00 | NF | NF |

Those with z-scores >2 or <-2 are considered significant.
