# Supplementary material for: Unveiling Integrated Functional Pathways Leading to Enhanced Respiratory Disease Associated With Inactivated Respiratory Syncytial Viral Vaccine
Source: Front Immunol. 2019 Mar 29;10:597. doi: 10.3389/fimmu.2019.00597 (PMC6449435; doi:10.3389/fimmu.2019.00597)
Supplement: Supplementary file 3 [file Table_3.docx]

Supplementary Table3: Disease or Functional Annotations associated with Hematological System Development and Function in FI-RSV vaccinated Cotton Rats challenged with RSV.

|  | **Diseases or Functions Annotation** | **p-Value** | **Activation z-score** | **# Molecules** |
| --- | --- | --- | --- | --- |
| **Functional Annotations related to hematological homeostatic imbalance** | quantity of blood cells | 8.37E-06 | 1.48 | 36 |
|  | activation of blood cells | 6.75E-08 | 2.37 | 34 |
|  | differentiation of blood cells | 4.35E-04 | 1.33 | 27 |
|  | aggregation of blood cells | 9.50E-12 | 1.49 | 23 |
|  | aggregation of blood platelets | 5.82E-11 | 0.95 | 20 |
|  | hemostasis | 6.64E-09 | 1.72 | 18 |
|  | coagulation of blood | 1.65E-08 | 0.95 | 16 |
|  | accumulation of blood cells | 1.59E-05 | 0.72 | 15 |
|  | blood pressure | 3.33E-04 | 0.09 | 14 |
|  | adhesion of blood platelets | 1.08E-04 | 1.72 | 6 |
|  | shape change of blood platelets | 4.20E-06 | 1.52 | 6 |
|  | fibrinolysis | 4.60E-06 | ND | 5 |
|  | cell spreading of blood platelets | 1.54E-05 | 1.17 | 5 |
|  | binding of blood platelets | 5.35E-04 | 0.46 | 5 |
|  | phagocytosis of blood platelets | 1.08E-04 | ND | 3 |
| **Functional Annotations related to cellular movement and infiltration** | cell movement of leukocytes | 7.17E-10 | 2.62 | 39 |
|  | cell movement of phagocytes | 3.25E-11 | 2.55 | 34 |
|  | cell movement of myeloid cells | 2.66E-11 | 2.07 | 34 |
|  | activation of leukocytes | 3.89E-08 | 2.57 | 33 |
|  | quantity of leukocytes | 6.57E-05 | 0.89 | 31 |
|  | proliferation of immune cells | 1.09E-04 | 1.27 | 28 |
|  | cell movement of granulocytes | 5.42E-11 | 2.90 | 27 |
|  | cell movement of neutrophils | 1.30E-12 | 2.85 | 26 |
|  | proliferation of lymphocytes | 1.46E-04 | 0.98 | 26 |
|  | cellular infiltration by leukocytes | 4.82E-09 | 1.17 | 25 |
|  | activation of myeloid cells | 1.12E-09 | 2.44 | 24 |
|  | activation of phagocytes | 4.93E-09 | 2.40 | 24 |
|  | cell proliferation of T lymphocytes | 3.22E-05 | 1.09 | 24 |
|  | chemotaxis of leukocytes | 5.66E-09 | 2.86 | 23 |
|  | chemotaxis of phagocytes | 4.61E-10 | 2.68 | 22 |
|  | chemotaxis of myeloid cells | 3.98E-10 | 2.67 | 22 |
|  | adhesion of immune cells | 7.03E-07 | 3.17 | 20 |
|  | cell movement of antigen presenting cells | 2.25E-06 | 0.68 | 19 |
|  | cell movement of macrophages | 1.36E-07 | 0.08 | 18 |
|  | quantity of phagocytes | 7.14E-05 | -0.43 | 18 |
|  | recruitment of leukocytes | 8.78E-06 | 2.36 | 17 |
|  | activation of antigen presenting cells | 7.56E-06 | 2.28 | 17 |
|  | quantity of myeloid cells | 1.84E-05 | -1.09 | 17 |
|  | quantity of granulocytes | 1.43E-05 | -0.82 | 16 |
|  | activation of macrophages | 3.07E-06 | 2.09 | 15 |
|  | cellular infiltration by granulocytes | 9.43E-07 | 1.45 | 15 |
|  | quantity of B lymphocytes | 5.24E-04 | 2.16 | 14 |
|  | recruitment of phagocytes | 1.70E-05 | 1.73 | 14 |
|  | infiltration by neutrophils | 1.82E-07 | 1.69 | 14 |
|  | chemotaxis of neutrophils | 7.15E-08 | 1.64 | 14 |
|  | accumulation of leukocytes | 3.73E-05 | 0.49 | 14 |
|  | function of myeloid cells | 2.50E-05 | ND | 13 |
|  | migration of phagocytes | 2.36E-04 | 2.02 | 13 |
|  | adhesion of granulocytes | 2.85E-08 | 2.56 | 12 |
|  | adhesion of phagocytes | 9.56E-07 | 2.52 | 12 |
|  | recruitment of myeloid cells | 1.13E-04 | 1.88 | 12 |
|  | accumulation of myeloid cells | 6.25E-06 | 1.35 | 12 |
|  | quantity of neutrophils | 8.17E-05 | -0.50 | 12 |
|  | adhesion of neutrophils | 1.48E-08 | 2.40 | 11 |
|  | activation of granulocytes | 5.17E-07 | 2.07 | 11 |
|  | recruitment of granulocytes | 2.67E-04 | 1.82 | 11 |
|  | cellular infiltration of phagocytes | 1.09E-04 | 0.64 | 11 |
|  | migration of myeloid cells | 1.52E-04 | 2.31 | 10 |
|  | engulfment of myeloid cells | 6.49E-05 | 1.83 | 10 |
|  | activation of neutrophils | 3.51E-07 | 1.76 | 10 |
|  | cellular infiltration by macrophages | 1.30E-04 | -0.18 | 10 |
|  | morphology of myeloid cells | 3.05E-04 | ND | 9 |
|  | migration of granulocytes | 2.05E-04 | 2.70 | 9 |
|  | adhesion of mononuclear leukocytes | 1.81E-04 | 1.77 | 9 |
|  | binding of phagocytes | 3.22E-05 | 1.71 | 9 |
|  | phagocytosis of myeloid cells | 2.23E-04 | 1.63 | 9 |
|  | immune response of neutrophils | 1.12E-06 | 0.96 | 9 |
|  | binding of professional phagocytic cells | 1.91E-04 | 2.07 | 8 |
|  | cell viability of myeloid cells | 2.42E-05 | 1.97 | 8 |
|  | recruitment of macrophages | 5.89E-05 | 1.82 | 8 |
|  | phagocytosis of neutrophils | 2.11E-07 | 1.45 | 8 |
|  | function of neutrophils | 1.04E-04 | ND | 7 |
|  | chemotaxis of macrophages | 4.09E-04 | 2.00 | 7 |
|  | binding of neutrophils | 1.09E-06 | 1.73 | 7 |
|  | accumulation of granulocytes | 4.98E-04 | -0.22 | 7 |
|  | quantity of eosinophils | 4.74E-04 | -0.64 | 7 |
|  | cell spreading of neutrophils | 5.96E-08 | 1.66 | 6 |
|  | activation of mast cells | 3.03E-04 | 1.40 | 6 |
|  | respiratory burst of neutrophils | 4.20E-06 | -0.05 | 6 |
|  | cell-cell adhesion of leukocytes | 2.82E-04 | ND | 4 |
|  | morphology of granulocytes | 4.69E-04 | ND | 4 |
|  | differentiation of bone marrow-derived macrophages | 3.67E-04 | 0.96 | 4 |
|  | abnormal morphology of neutrophils | 1.08E-04 | ND | 3 |

Results are presented with the p-value of overlap which predicts the likelihood that the disease and/or functional outcome occurs, along with the corresponding z-score showing the predicted activation state of each disease or functional annotation, and the number of molecules in the dataset associated with the hematological system development of function. ND = Not determined.
