# Supplementary material for: Unveiling Integrated Functional Pathways Leading to Enhanced Respiratory Disease Associated With Inactivated Respiratory Syncytial Viral Vaccine
Source: Front Immunol. 2019 Mar 29;10:597. doi: 10.3389/fimmu.2019.00597 (PMC6449435; doi:10.3389/fimmu.2019.00597)
Supplement: Supplementary file 4 [file Image_1.pdf]

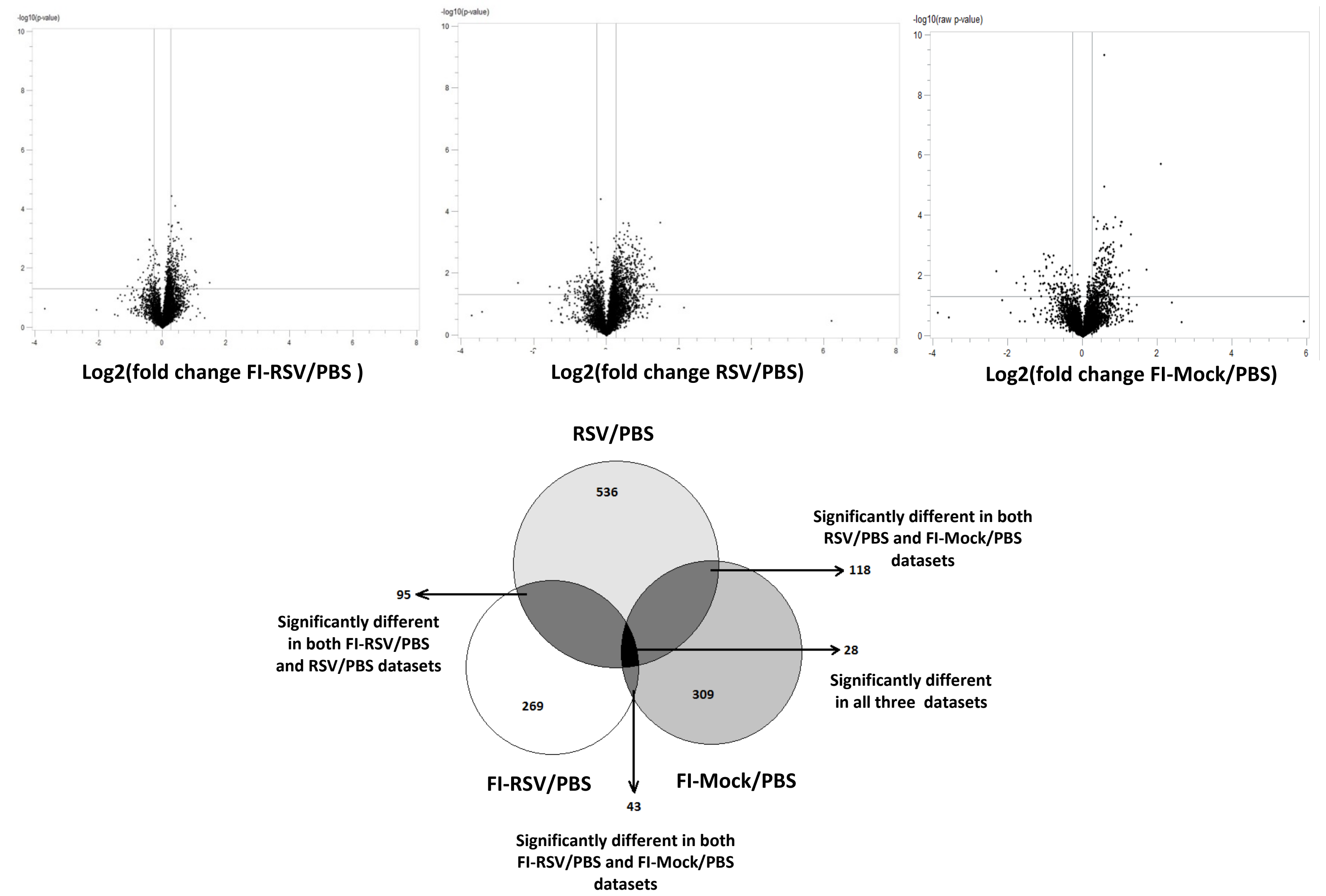

Supplementary Figure1: Volcano plot illustrates significantly differentially abundant protein levels and Venn diagram show protein comparisons between FI-RSV, RSV, and FI-Mock. In volcano plot, points above the non-axial horizontal line represent the proteins with significantly different abundances based on the t-test statistics (alpha-level=0.05). Points to the left of the left-most non-axial vertical line denote protein fold change of less than 1/1.2, while points to the right of the right-most non-axial vertical line denote protein fold change of greater than 1.2. The values of fold change allow direct comparison of protein abundance. The Venn diagram shows the number of differentially abundant proteins in the respective groups and between the groups. A total of 5069 proteins were

detected in the FI-RSV, RSV, and FI-Mock groups. Further analyses indicated that FI-RSV group had 269 significant differentially expressed proteins from PBS, the RSV group had 536 proteins, and FI-Mock had 309 proteins. There are 95 differentially expressed proteins shared between the FI-RSV and the RSV groups, 118 proteins between RSV group and the FI-Mock group, and 43 proteins between FI-RSV and FI-Mock. Overall, 28 differentially expressed proteins were shared by all groups.
